# Supplementary material for: Serum Level of Tumor-Overexpressed AGR2 Is Significantly Associated with Unfavorable Prognosis of Canine Malignant Mammary Tumors
Source: Animals (Basel). 2021 Oct 9;11(10):2923. doi: 10.3390/ani11102923 (PMC8532596; doi:10.3390/ani11102923)
Supplement: Supplementary file 1 [file animals-11-02923-s001.zip › animals-1371608-supplementary.pdf]

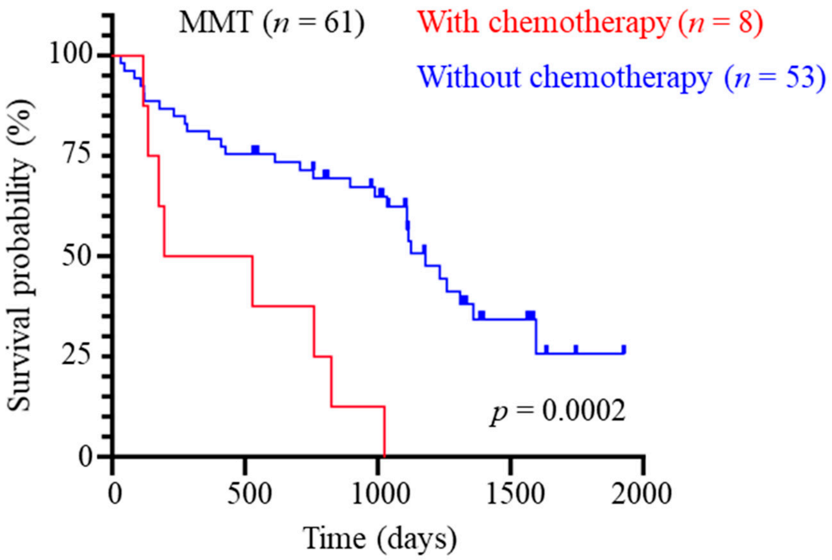

Chemotherapy	eAGR2 concentration (μg/mL)	
	Mean ± SD	Median (range)
Yes (n = 8)	7.99 ± 3.53	8.58 (2.39 – 12.49)
No (n = 53)	5.53 ± 2.78	4.91 (1.76 – 15.55)

Figure S1. Comparison of overall survival between MMT dogs with and without chemotherapy after surgery. The Kaplan-Meier survival plot depicts differences in survival probability between the chemotherapy-administered and the chemotherapy-free groups in 61 MMT cases. Statistical significance was determined by the log-rank test. Data of serum eAGR2 concentration in two groups are shown in the bottom table.

(A)

Figure 2A

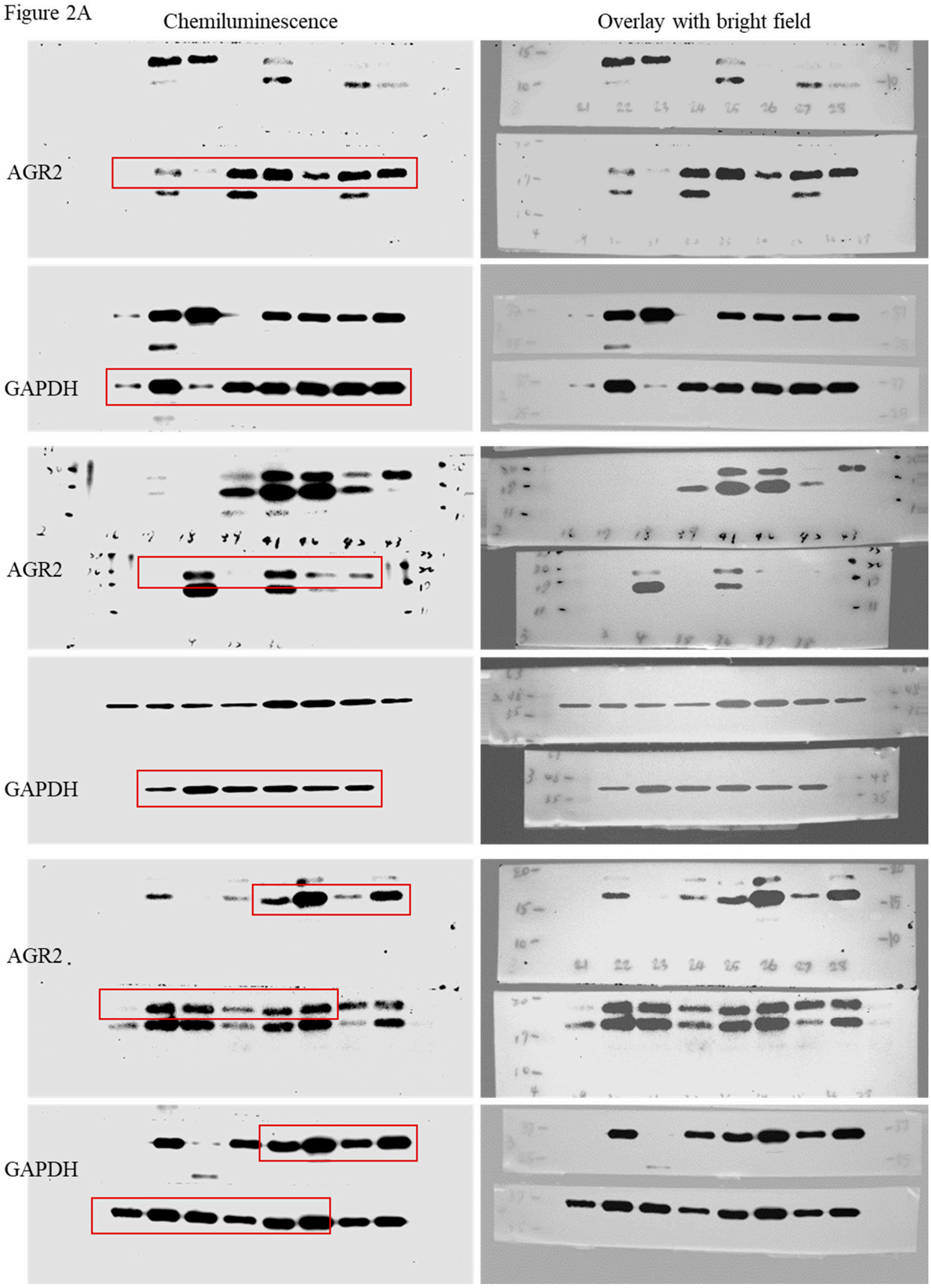

Chemiluminescence
Overlay with bright field
AGR2
GAPDH
AGR2
GAPDH
AGR2
GAPDH

(B)

Figure 3B

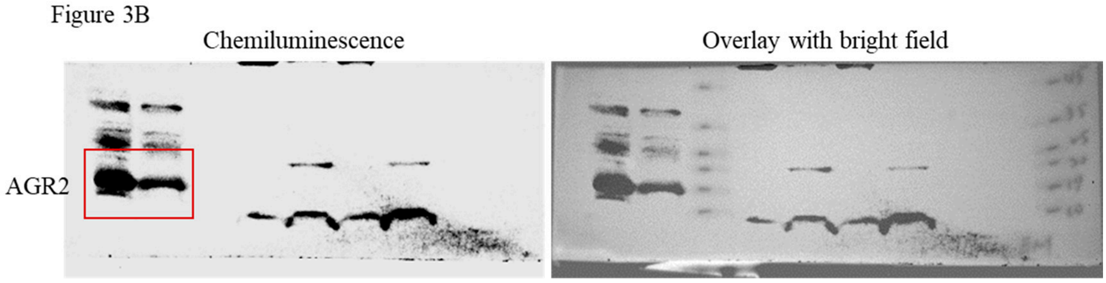

(C)

Figure 4B

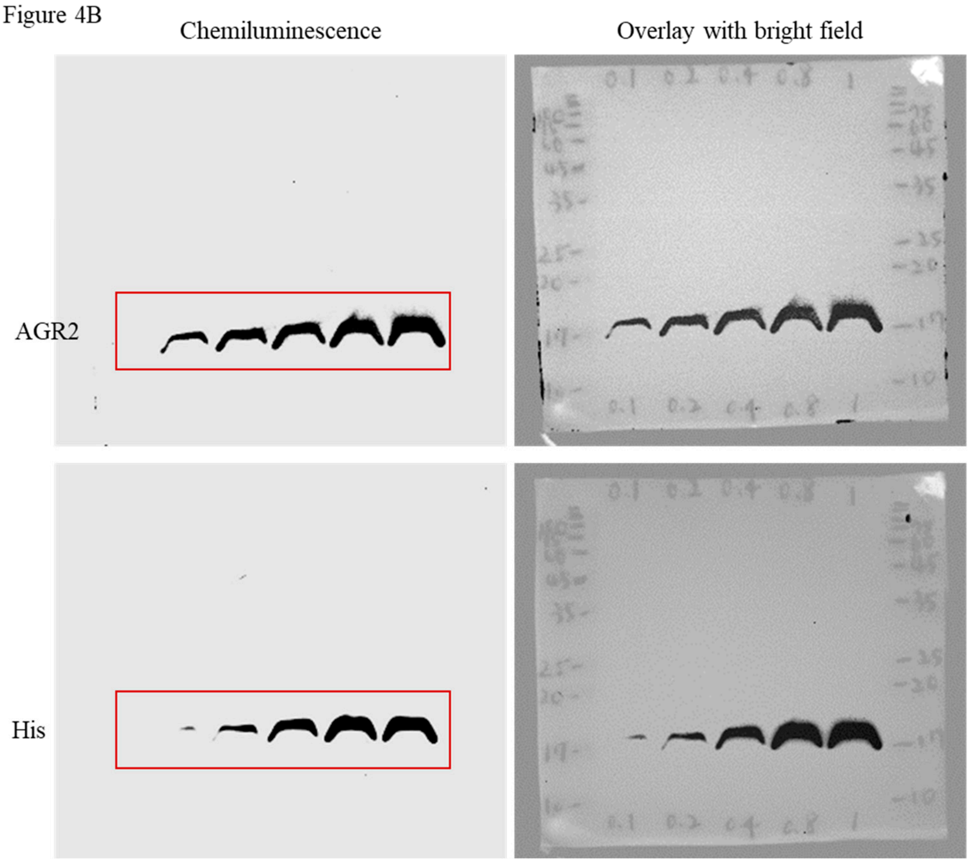

Figure S2. Original immunoblotting data in this study. (A) Figure 2A; (B) Figure 3B; (C) Figure 4B.
